# A very easy technique of stenting for laparoscopic pyeloplasty: penbegul intravenous cannula (PICA) technique

**DOI:** 10.1590/S1677-5538.IBJU.2018.0303

**Published:** 2019

**Authors:** Necmettin Penbegul, Murat Atar, Cem Alan, Yasar Bozkurt, Namik Kemal Hatipoglu

**Affiliations:** 1 Department of Urology, VM Medical Park Bursa Hospital, Bursa, Turkey; 2 Department of Urology, Dicle University School of Medicine, Diyarbakir, Turkey

**Keywords:** Stents, Laparoscopy, Surgical Procedures, Operative

## Abstract

**Introduction:**

Double-J stent insertion during laparoscopic pyeloplasty is a difficult and time-consuming process and several techniques were defined to perform a double-J stent with an antegrade approach. In this study we present the technique (PICA) of antegrade double-J placement during laparoscopic pyeloplasty by using 14 gauge intravenous cannula.

**Surgıcal technıque:**

After we complete the suturing of the posterior wall of the anastomosis during laparoscopic pyeloplasty, we first puncture the abdominal wall with a 14-gauge “intravenous cannula” from a location that provides most suitable angle for inserting the double-J stent into the ureter. We remove the metal needle of the cannula, and the sheath which has an inner diameter of 5.2F remains over the abdominal wall. The double J stent is then advanced from inside the cannula sheath to the intraperitoneal area; under laparoscopic imaging the stent is gently grasped at its distal end using an atraumatic laparoscopic forceps to insert it into the ureter. The stent is then pulled down to its proximal end, and after the guidewire is removed, the proximal end of the double-J stent is placed inside the renal pelvis with an atraumatic forceps. With this technique we can apply the double-J stent in just one step. Additionaly we can use a 14-gauge IV cannula sheath as a trocar when needed during laparoscopic pyeloplasty to retract an organ or reveal an anastomosis line.

**Comments:**

Our new technique of antegrade double-J placement during laparoscopic pyeloplasty by 14 gauge intravenous cannula sheath, is very easy and quick to perform.

## INTRODUCTION

Laparoscopic pyeloplasty usually includes double-J stenting to allow adequate urine drainage and suturing and to prevent recurrent strictures during anastomotic healing. It is still a controversial issue to insert a double-J stent using an antegrade vs. a retrograde approach during laparoscopic pyeloplasty (1). We prefer to perform applying a double-J stent via antegrate approach such as most of the authors. Sometimes double-J stent insertion during laparoscopic pyeloplasty, is a difficult and time-consuming process because of the flexibility and coiled distal ends of the stent itself, the limited bending angle of the laparoscopic instruments, and the narrow laparoscopic visual field (2). Several techniques were defined to perform a double-J stent with an antegrade approach during laparoscopic pyeloplasty. In these techniques usually authors insert a guidewire through the 14 gauge cannule, 18 gauge cannula, J-tube or a 5mm port into the ureter down to the bladder and then they advanced a double-J stent over the guidewire. In all these techniques double-J insertion is completed by multiple steps (3–5). Herein, we report our new technique of antegrade double-J placement during laparoscopic pyeloplasty by 14 gauge intravenous cannula sheath, which overcomes the limitations of other techniques and is very easy and quick to perform. Also, after double-J placement, intravenous cannula sheath can be used as a trocar to retract an organ or reveal an anastomosis line.

## SURGICAL TECHNIQUE

**Figure 1:**
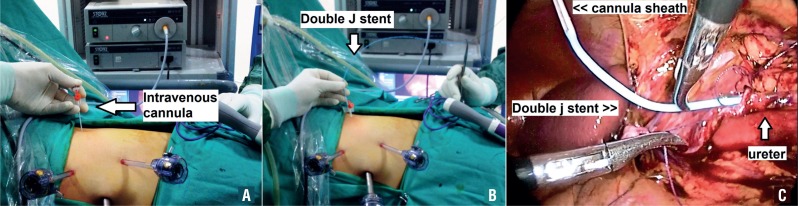
(A) IV cannula (14G) is performed from a suitable location. (B) The assembled stent is advanced from inside the IV cannula sheath. (C) Under laparoscopic imaging the stent is gently grasped at its distal end to insert it into the ureter.

Laparoscopic dismembered pyeloplasty is performed by transperitoneal technique in all patients at our institution. Usually three trocars are introduced with the telescope port located in an umbilical position. After pelvic reduction and the spatulation of the ureter, we place the first corner suture between the renal pelvis and the spatulated ureter. Next, we complete the suturing of the posterior wall of the anastomosis. At this stage we first puncture the abdominal wall with a 14-gauge “intravenous cannula” from a location that provides most suitable angle (mostly cephalad to the anastomotic site) for inserting the double-J stent into the ureter (Figure-1A). We remove the metal needle of the cannula, and the sheath which has an inner diameter of 5.2F remains over the abdominal wall. We assemble a double-J stent extracorporeally that is comprised of the closed end double-J stent (up to 4.8F caliber), a guide wire, a pusher, and a clip (Figure-1B). The assembled stent is then advanced from inside the IV cannula sheath to the intraperitoneal area; under laparoscopic imaging, the stent is gently grasped at its distal end using an atraumatic laparoscopic forceps to insert it into the ureter (Figure-1C). The stent is then pulled down to its proximal end, and after the guidewire is removed, the proximal end of the double-J stent is placed inside the renal pelvis with an atraumatic forceps. All these steps are completed in a few minutes (maximum 3, 5 minutes in last 10 patients). Unlike in the other techniques, we can apply the double-J stent in just one step. After we place the stent, we insert a plug at the external end of the sheath to prevent air leaks. An important component is that we never remove the sheath because we may be able to use this sheath during surgery as a trocar, a procedure that we will describe in the next paragraph.

**Figure 2:**
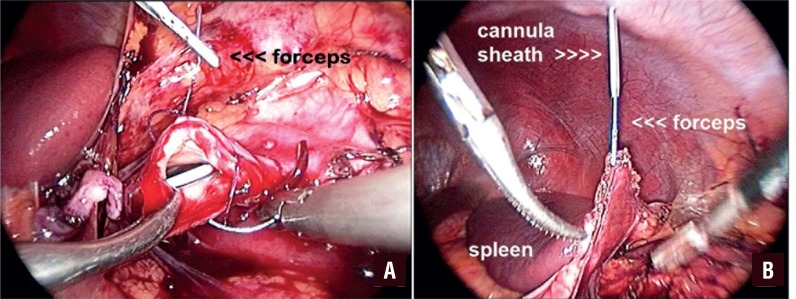
(A) A 5F forceps that is inserted inside of the IV cannula sheath is used to retract anastomosis line during suturing. (B) A 5F forceps that is inserted inside of the IV cannula sheath is used to retract spleen during left renal laparoscopic surgery. Video presentation: The video presentation shows details of antegrade double-J placement during laparoscopic pyeloplasty by 14 gauge intravenous cannula sheath, and also shows the use of intravenous cannula sheath as a trocar to retract an organ or reveal an anastomosis line during laparoscopic urologic surgery.

Laparoscopic renal surgery is usually performed with three trocars. However, sometimes a fourth trocar is needed for suction or retraction. In our technique, we use a 14-gauge IV cannula sheath as a trocar when needed during laparoscopic pyeloplasty, especially in pediatric patients. As we described in the previous paragraph, we place a 14-gauge IV cannula sheath for inserting the double-J stent, and after we have inserted the stent, we do not remove the sheath. During the later stages of the operation, we insert a forceps (up to a 5F caliber) when necessary from inside the sheath to retract an organ or reveal an anastomosis line (Figure-2A). These forceps are usually used with ureterorenoscopes or pediatric cystoscopes. Therefore, when an additional trocar is needed, we can almost always solve the problem with the IV cannula sheath that is already applied for the double-J stent insertion. Hereby; “kill two birds with one stone” as said in a Turkish proverb. This trocar caliber (2.2mm) is the smallest mentioned in the literature.

## COMMENTS

Endourologic surgery has witnessed incredible developments, particularly in the field of minimally invasive surgery, which includes percutaneous stone removal, transurethral treatment of urinary stones, laparoscopic surgeries, and robot-assisted laparoscopic surgeries. Based on these developments, a significant variety of the armamentarium has increased in urology practice. As a result, the costs of urologic management have exploded which had a negative impact on future developments. Despite these technological innovations, some simple instruments that are still very useful in daily practice may be overlooked. Many authors have described simple solutions that can be carried out during minimally invasive surgery that lower the cost of treatments. For this purpose we have a simple and easy suggestion by this study.

Intravenous (IV) cannulas are cheap, small, and readily available catheters that are mostly used in daily medical practice for peripheral venous access for the administration of intravenous fluids and medications and also for obtaining blood samples. Such devices are available in various gauges (16-24 gauge), lengths (25-45mm), compositions, and designs. IV cannulas consist of a transparent sheath, a metal needle, and a plug at the end of the metal needle. In addition to the traditional purposes in our urologic practice, we used IV cannulas in several pediatric endourologic surgeries (access needle during pediatric PNL, microsheath during pediatric microperc, renal drainage during pediatric microperc, microsheath during pediatric percutaneous cystolithotripsy). Additionally, we use IV cannula as a trocar for double-J catheter insertion and also for retraction during laparoscopic pyeloplasty with this technique.

There are several methods in the literature that use either antegrade or retrograde approaches. In techniques that employ an antegrade approach, surgeons firstly puncture the abdominal wall with a cannula or an access needle to advance a guide wire into the ureter and then remove the cannula or needle and lastly insert the double stent over the guidewire. However, in our technique, after we puncture the abdominal wall with a 14-gauge IV cannula at an appropriate location, we insert an assembled double-J stent (stent + guidewire + pusher + clip) inside the cannula and through the ureter in a single step. As a departure from the other techniques, we do not remove the cannula because of the possibility that it will be required for another situation (manipulation of the stent, reapplication of the stent, etc.). Finally, we can use IV cannulas during laparoscopic surgery as additional trocars. We do not remove the IV cannula during laparoscopic pyeloplasty and if needed, we insert a forceps inside this cannula to hold the tip of the suture during the suturing of an anastomosis. During both adult and pediatric laparoscopic surgery, we use 14-gauge IV cannulas for slight retraction if needed (Figure-2B). Thus, the cannula prevents the use of an additional trocar just for simple retraction. At the end of the surgery, the cannula leaves no visible scarring. As far as we are aware, this is the smallest trocar defined in the literature.

In conclusion, an IV cannula is the smallest Amplatz sheath, is the smallest access needle, can act as the smallest laparoscopic trocar, and is the cheapest instrument by far for pediatric endourology. Despite its small size, it can achieve great things. In brief, our new technique of antegrade double-J placement during laparoscopic pyeloplasty by 14 gauge intravenous cannula sheath is very easy and quick to perform.

## ABBREVIATIONS

IV: = Intravenous
